# LOXL2 promotes aggrecan and gender-specific anabolic differences to TMJ cartilage

**DOI:** 10.1038/s41598-020-77178-9

**Published:** 2020-11-19

**Authors:** Mustafa M. Tashkandi, Saqer F. Alsaqer, Thabet Alhousami, Faiza Ali, Yu-Chiao Wu, Jennifer Shin, Pushkar Mehra, Larry M. Wolford, Louis C. Gerstenfeld, Mary B. Goldring, Manish V. Bais

**Affiliations:** 1 Department of Translational Dental Medicine, Boston University Henry M. Goldman School of Dental Medicine, 700 Albany Street; W-202B, Boston, MA 02118 USA; 2 Department of Oral and Maxillofacial Surgery, Boston University Henry M. Goldman School of Dental Medicine, 100 East Newton Street, Boston, MA 02118 USA; 3 Texas A&M University College of Dentistry, Baylor University Medical Center, Dallas, TX USA; 4 Department of Orthopedic Surgery, School of Medicine, Boston University, Boston, MA USA; 5 Hospital for Special Surgery Research Institute, Department of Cell and Developmental Biology, Weill Cornell Medical College, New York, NY 10021 USA

**Keywords:** Diseases, Rheumatology

## Abstract

In the United States, 5–12% of adults have at least one symptom of temporomandibular joint (TMJ) disorders, including TMJ osteoarthritis (TMJ-OA). However, there is no chondroprotective agent that is approved for clinical application. We showed that LOXL2 is elevated in the regenerative response during fracture healing in mice and has a critical role in chondrogenic differentiation. Indeed, LOXL2 is an anabolic effector that attenuates pro-inflammatory signaling in OA cartilage of the TMJ and knee joint, induces chondroprotective and regenerative responses, and attenuates NF-kB signaling. The specific goal of the study was to evaluate if adenoviral delivery of LOXL2 is anabolic to human and mouse TMJ condylar cartilage in vivo and evaluate the protective and anabolic effect on cartilage-specific factors. We employed two different models to assess TMJ-OA. In one model, clinical TMJ-OA cartilage from 5 different samples in TMJ-OA cartilage plugs were implanted subcutaneously in nude mice. Adenovirus LOXL2 -treated implants showed higher mRNA levels of LOXL2, ACAN, and other anabolic genes compared to the adenovirus-Empty-treated implants. Further characterization by RNA-seq analysis showed LOXL2 promotes proteoglycan networks and extracellular matrix in human TMJ-OA cartilage implants in vivo. In order to evaluate if LOXL2-induced functional and sex-linked differences, both male and female four-month-old chondrodysplasia (Cho/+) mice, which develop progressive TMJ-OA due to a point mutation in the *Col11a1* gene, were subjected to intraperitoneal injection with Adv-RFP-LOXL2 every 2 weeks for 12 weeks. The data showed that adenovirus delivery of LOXL2 upregulated LOXL2 and aggrecan (*Acan*), whereas MMP13 expression was slightly downregulated. The fold change expression of *Acan* and *Runx2* induced by Adv-RFP-LOXL2 was higher in females compared to males. Interestingly, Adv-RFP-LOXL2 injection significantly increased *Rankl* expression in male but there was no change in females, whereas *VegfB* gene expression was increased in females, but not in males, as compared to those injected with Adv-RFP-Empty in respective groups. Our findings indicate that LOXL2 can induce specifically the expression of Acan and other anabolic genes in two preclinical models in vivo. Further, LOXL2 has beneficial functions in human TMJ-OA cartilage implants and promotes gender-specific anabolic responses in Cho/+ mice with progressive TMJ-OA, suggesting its merit for further study as an anabolic therapy for TMJ-OA.

## Introduction

Temporomandibular joint (TMJ) disorders include a group of complex and poorly understood conditions characterized by orofacial pain, popping sounds, and restricted joint movement leading to anatomical changes. The female-to-male ratio of TMJ disorder incidence ranges from 3:1 to 9:1^1–3^. TMJ osteoarthritis (OA) is a degenerative disease that affects both cartilage and subchondral bone. TMJ-OA is the most prevalent of TMJ disorders^4^, which affects 5 to 12 percent of the population and associated with an estimated annual cost of $4 billion^5^. However, there is no chondroprotective agent is approved for clinical application. A clinical study showed significant differences in OA-associated inflammatory protein expression between TMJ and knee joint OA, suggesting differences in pathophysiology at these sites^6^. TMJ has fibrocartilage with functional and pathological differences^7–11^ compared to cartilage in other joints. Defective TMJ cartilage can undergo regeneration following stimulation on chondroprogenitor or mesenchymal stem cells by specific molecular cues^12–14^. A potential stimulatory agent is lysyl oxidase like-2 (LOXL2). Lysyl oxidase (LOX) family members, LOXL1-4, are copper-dependent amine oxidases with intracellular and extracellular roles in extracellular matrix (ECM) remodeling, collagen crosslinking, and cell differentiation^15,16^.

We previously showed that the LOXL2 is elevated in the regenerative tissues during fracture healing in mice compared to other LOX isoforms^17^. Subsequently, we determined that LOXL2 expression is critical for chondrogenic differentiation and proteoglycan deposition in a chondrocyte culture model^18^. More recently, we identified LOXL2 as an anabolic effector that attenuates pro-inflammatory signaling in OA cartilage of the TMJ and knee joint in vitro and in vivo^19,20^. We developed a model in which human TMJ cartilage was implanted in nude mice in combination with a specialized matrix of the basement membrane. This commercially available matrix, Geltrex, helps to maintain pluripotent stem cells without adversely affecting chondrogenic differentiation^21^. Previous studies also used the combination of adenovirus with Geltrex to study endochondral ossification during porcine cartilage repair in vivo^22^. Here, we investigated whether LOXL2 could influence the anabolic responses in human and mouse TMJ-OA cartilage, and promote expression of anabolic genes and proteoglycans, In addition, we addressed gender-specific differences in the anabolic response, which had not been addressed previously, because this is critical to the understanding of the application of LOXL2 for future translation.

Aggrecan is almost 10% of articular cartilage^23^, endowing it with the ability to withstand compressive loads due to its structural properties conferred by the glycosaminoglycan (GAG) side chains of chondroitin sulfate and keratan sulfate chains arranged along with the aggrecan core protein. Aggrecan is synthesized intracellularly as GAG-rich proteoglycan monomers. The monomers are then assembled within the ECM in the form of large proteoglycan aggregates attached to long polymers of hyaluronic acid in a noncovalent manner to the G1 domains of aggrecan^24^ via link protein, which stabilizes the interaction^25,26^.

The Cho/+ OA mouse develops progressive OA-associated changes with age due to haploinsufficiency of type XI collagen, the expression of which is reduced in human articular cartilage of older individuals^27^. Data from human and animal experiments showed that decreased type XI collagen in cartilage may be one of the initiating factors in the pathogenesis of TMJ-OA and knee-OA^28,29^. A single-nucleotide deletion in Cho/+ mice results in premature termination of the α1 chain of type XI collagen^30^. This leads to the progressive knee-OA starting at 3 months of age, with increasing severity up to 15 months of age^28,29,31,32^. The differences between wild type and Cho−/+ mice were well characterized in earlier studies, which demonstrated progressive changes in the TMJ articular cartilage with aging^31,32^. At the age 3 months, the proteoglycans in wild type mice are abundant in the middle and deep zones of articular cartilage in the TMJ, whereas in Cho/+ mice the proteoglycans are more diffuse. The depletion of proteoglycans from the entire TMJ cartilage and flattening of the condylar head become evident at 6 months, and fibrillation is seen at 9 months in the TMJ of Cho−/+ mice^29^.

In the present study, we used TMJ-OA cartilage/Geltrex constructs that were implanted subcutaneously in nude mice and transduced with adenoviral LOXL2. This allowed us to assess the efficacy of the LOXL2 in enhancing an anabolic response specifically in human cells. Since the Cho/+ mouse develops progressive TMJ-OA changes -associated with age, this model permitted us to assess the ability of LOXL2 to attenuate the progression of OA and evaluate the gender-specific differences. Using a combination of histological, microscopic, and molecular assessments in both models, we observed that adenoviral LOXL2 delivery in vivo produces both chondroprotective and anabolic effects in chondrocytes, suggesting therapeutic implications for the potential efficacy of mediators of LOXL2 in treating TMJ-OA.

## Materials and methods

### Use of human tissues and animal experiments

Human tissue samples were collected with informed consent and experiments were conducted in accordance with relevant guidelines and regulations of Boston University Institutional Review Board (IRB; approval number H33300) with age group 45–65 yrs. The study on humans was approved by the corresponding ethics committee (IRB; approval number H33300). All mouse experiments were performed with guidance, regulations, and approval of Boston University Institutional Animal Care and Use Committee (IACUC; approval number AN-15387. The preclinical animal study conformed to the ARRIVE guidelines.

### Preparation of adenovirus

As described in our earlier studies^19,20^, adenoviruses for LOXL2 expression co-expressing Red Fluorescent Protein (RFP) was used for verification of virus transduction and efficient of delivery Ad-CMV-RFP-CMV-hLOXL2-His; referred to as Ad-RFP-LOXL2), and the empty vector control (Ad-RFP-Empty) were custom synthesized (ADV-214438) by Vector Biolabs, PA. These adenovirus particles were amplified in 293 T cells and quantified using an adenovirus quantification kit (Cell Biolabs).

### Cho/+ mouse experiments

The Cho/+ mice were obtained originally from Dr. Yefu Li, Harvard Medical School, bred with C57/BL6 to maintain a colony of heterozygous mice, and genotyped by PCR.16-week-old Cho/+ mice were divided into 2 groups: (1) Adv-RFP-Empty was administered to 14 male and 14 female mice; and (2) Adv-RFP-LOXL2 was administered to 14 male and 14 female mice every 2 weeks by intraperitoneal injection (50 µL; concentration 10^13^ infectious particles/mL) for 12 weeks. TMJ condylar cartilage samples were harvested and subjected to total RNA extraction, RT-qPCR, and histology analysis.

### Human OA tissues from TMJ joints

Anonymized pathological waste TMJ tissues were obtained from Boston University Medical Center, Boston, MA, through IRB approval (H33300). The exclusion criteria included a history of any rheumatic disease or cancer. The samples were selected based on clinical features such as TMJ pain and joint mobility and then confirmed as TMJ-OA by pathological diagnostic findings from TMJ tissue sections at the Department of Pathology, Boston University.

These freshly isolated TMJ condylar cartilage tissues were divided into 2 parts: (1) for preparation of fixed and stained sections for histological characterization or (2) for preparation of biopsy punches (4-mm^3^) for nude mice implant studies, starting from the superficial layer and extending towards proteoglycan layer in order to maintain uniformity in the implant size and shape as described below.

### RNA isolation and analysis

Total RNA was extracted by the Trizol protocol according to the manufacturer’s instructions (Qiagen) as described earlier^19,20^. RT-qPCR analysis was performed using TaqMan gene expression assays from Life Technologies, according to a standard protocol^33^.

### Histology and immunostaining

TMJ from Cho/+ mice or cartilage implants were paraffin-embedded, decalcified, and subjected to histological analysis and immunostaining. Safranin-O/Fast Green (American Mastertek Inc.) staining was performed as described in earlier studies^19^ and Sirius red (American Mastertek Inc.) staining was performed as per the instructions from the manufacturer. Immunostaining with specific antibodies to detect RFP, LOXL2, Acan, and MMP13 (Abcam), was performed after de-paraffinization of sections and visualized with HRP-linked anti-rabbit antibody for imaging. For immunofluorescence analysis,de-paraffinized section were subjected to antigen retrieval using 0.4% pepsin, washed and incubated with primary antibody (anti-LOXL2 or anti-RFP or isotype control for overnight, followed by washing and secondary antibody incubation for 50 min. Next after washing subjected to DAPI staining for 10 min, washed again and subjected to Sudan black block background for 5minutes and finally washed in running water and imaged with fluorescence microscopy.

### Modified Mankin scoring

A modified Mankin scoring system was followed, as described in earlier studies^29,34^. The extent of OA histopathology was quantified from Safranin-O/Fast green staining sections by the sum of scores of the following four criteria: surface fissuring (0–3), pericellular matrix staining (0–2), the spatial arrangement of chondrocytes (0–3), and interterritorial matrix staining (0–3). The scores of each group were averaged and compared for statistical significance; the higher the score implies more advanced OA histopathology. The lower (200X) and higher (730X) magnification are presented to demonstrate all of the above four criteria with extensive histological analysis.

### Immunofluorescence analysis of LOXL2

Paraffin-embedded tissue sections were labeled with. To detect LOXL2 expression, the tissues were incubated with rabbit anti-LOXL2 (GeneTex, Inc) or its isotype control antibody and detected with anti-rabbit biotin followed by streptavidin-conjugated Texas red. For immunofluorescence analysis, anti-rabbit IgG conjugated to Alexa 488antibody Anti-fade reagent with DAPI was added to all samples before imaging. Image analysis was performed using Zeiss LSM viewer and Image J software (NIH, USA)^19,20^.

### Human cartilage implants in nude mice

TMJ-OA condylar cartilage explants (4-mm^3^) were generated using biopsy punch from the. These implants were surgically implanted subcutaneously in the backs of nude mice (3 implants/mouse). These explants were implanted with specialized matrix (Geltrex; ThermoFisher Scientific, Inc) as a basement membrane to promote a chondrogenic environment^21,22^. Geltre matrix is composed of laminin, collagen IV, entactin, and heparan sulfate proteoglycans. Geltrex is lactose dehydrogenase-elevating virus (LDEV)-free and basement membrane matrix with very low growth factor activity. Geltrex helps to maintain pluripotent stem cells and does not adversely affect chondrogenic differentiation^21^. Furthermore, the combination of adenovirus with Geltrex was previously used to study endochondral ossification during porcine cartilage repair in vivo^22^. At 7 days after implantation of TMJ-OA cartilage/Geltrex surgical constructs, the nude mice were treated locally near the implant by weekly injection of a 100-µL suspension of adenovirus, Adv-RFP-LOXL2 or Adv-RFP-Empty (n = 5/ condition), for 6 weeks. The mice were killed and implants were used for RNA isolation, RNA-seq, RT-qPCR, and histological analysis.

### RNA-seq analysis

RNA-seq library preparation was performed using a kit (Illumina, Inc) from 100 ng of total RNA and subjected to sequencing using an Illumina 500 sequencer with 24 million reads per sample. In order to evaluate the specific expression of human TMJ cartilage-related gene expression, the RNA Express Workflow and data filtering were run twice: once using human genome hg19 and once using mouse genome mm10. FASTQ files were aligned to human genome build hg19 and normalized to produce gene-level counts using version 1.1.0 of the Illumina BaseSpace RNA Express application, which uses STAR^35^ (version 2.4.0j), the DESeq2 R package^36^, and iGenomes RefSeq annotation build 2013–03-06. TruSeq adapters were trimmed prior to alignment and reads were counted in a stranded manner. The RNA Express workflow was repeated using mouse genome build mm10 to estimate the number of mouse reads contaminating the sample. Separately, SAMtools^37^ (version 1.4) was used to count reads aligning uniquely as a pair to the mitochondrial chromosome or to contig GL000220.1, and HTSeq^38^ (version 0.6.1p1, run under Python 2.7.3) was used to count those in the latter group that also aligned to the sense or antisense strand within locus *RNA45S5* (GL000220.1:105,424–118,780).

For Principal Component Analysis (PCA) the log2 transformation of the number of counts per gene normalized to the millions of read pairs mapping uniquely to RefSeq exons and added to a pseudocount of 1 was performed using the *prcomp* R function with log2(RPM + 1) expression values as described. The log2(RPM + 1) values were normalized across all samples to a mean of zero and a standard deviation of one. PCA was performed and output compiled using the R environment for statistical computing (version 2.15.1).

Benjamini–Hochberg False Discovery Rate (FDR) correction was also applied to obtain FDR-corrected *p* values (*q* values), which represent the probability that a given result is a false positive based on the distribution of all *p* values on the array. Corrected/adjusted *p* values such as the FDR *q* are the best measure of significance for a given test when many hypotheses (genes) are tested at once. The FDR *q* values were computed only across the genes that were determined to be reasonably well-expressed (a call of "OK"), as genes with low overall expression are more strongly affected by random technical variation and more likely to produce false-positive results. The estimated inter-group fold changes, converted from DESeq2 output (log2-transformed fold changes) to signed, linear fold changes (e.g., + 2 = twofold higher in LOXL2 than in control; 1 = no change; -2 = twofold lower in LOXL2 than in control). A Wald statistic (analogous to a *t* statistic) derived from the DESeq2 output (log2FoldChange/lfcSE, i.e., the estimated log2(fold change) divided by the standard error). The number of counts for each gene across all samples laid over a colored representation (heatmap). The colors of the heatmap have been scaled using log2(RPM + 1) values, so that red and blue indicate log2(RPM + 1) values ≥ 2 standard deviations above and below, respectively, the row-wise mean (white) computed across all samples.

Gene Set Enrichment Analysis (GSEA) (version 2.2.1)^39^ was used to identify biological terms, pathways and processes that are coordinately up- or down-regulated within each pairwise comparison. The genes included in the iGenomes RefSeq annotation that corresponded to exactly one human Entrez Gene identifier were ranked according to the Wald statistic computed between the LOXL2-treated and control groups within each species. Each ranked list was then used to perform pre-ranked GSEA analyses (default parameters with random seed 1234) using the Entrez Gene versions of the Hallmark, Biocarta, KEGG, Reactome, Gene Ontology (GO), and transcription factor and microRNA motif gene sets obtained from the Molecular Signatures Database (MSigDB), version 6.0^40^. The KEGG pathway analysis has been used for the analysis using the background and methods from the published studies^41–43^ Finally, selected genes showing relevance to human cartilage anabolic or catabolic response which are responsive to LOXL2 were validated by RT-qPCR analysis.

## Results

### LOXL2 promotes anabolic responses and aggrecan expression in TMJ-OA implants in vivo

**Figure 1:**
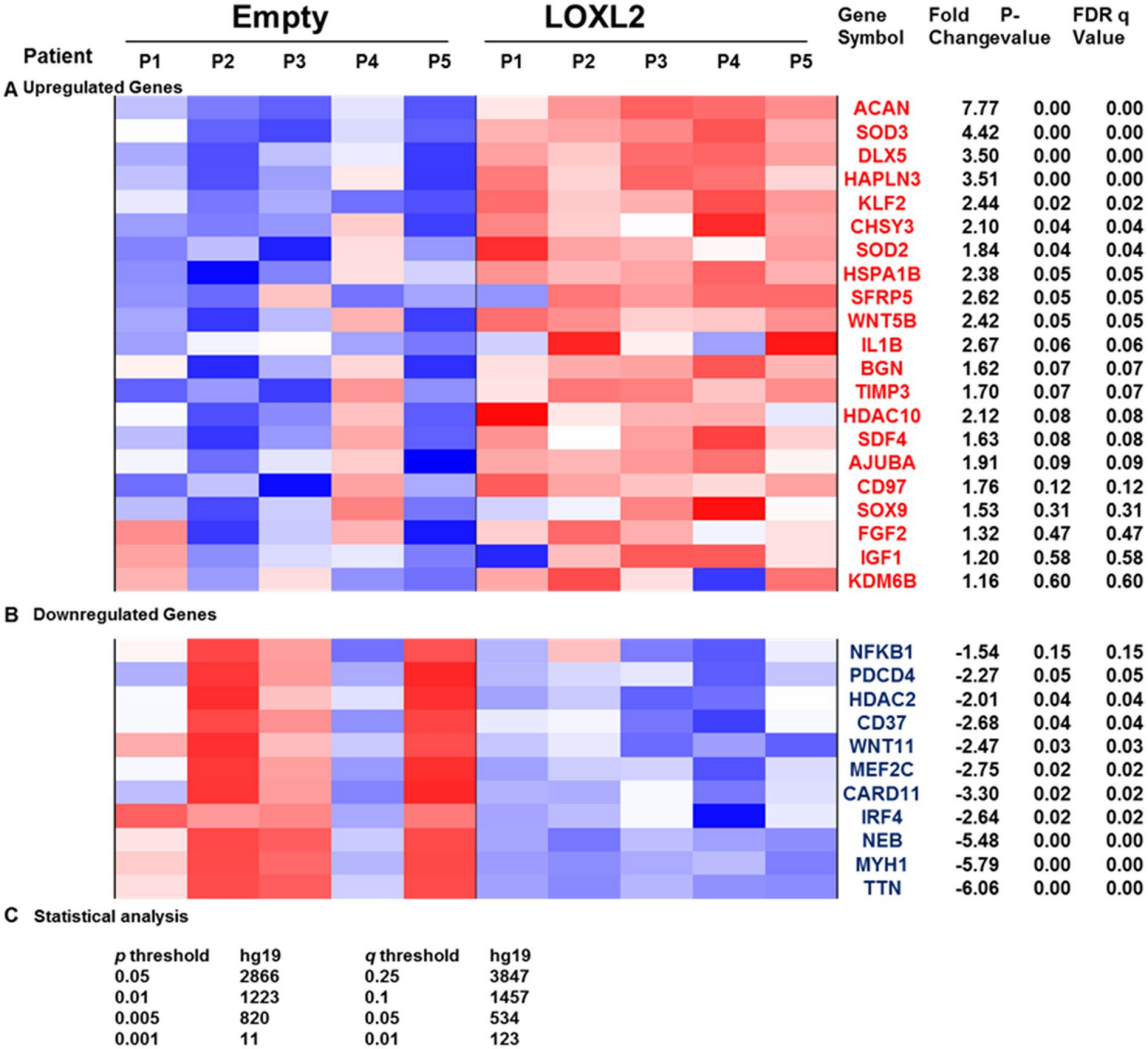
LOXL2 overexpression differentially regulates genes in human TMJ-OA cartilage implants in nude mice. Human TMJ-OA explants of cartilage/Geltrex were implanted into nude mice. P1 to P5 represent the human TMJ-OA cartilage explants from 5 different patients implanted in the respective mouse. These implants were treated in vivo every 2 weeks with injections of Ad-RFP-LOXL2 or Ad-RFP-Empty and total RNA extracts were subjected to global RNA-seq gene expression analysis. LOXL2 overexpression in TMJ-OA induced (**A**) upregulated gene signatures, (**B**) down-regulated gene signatures and (**C**) statistical analysis. The rows are sorted in descending order by Wald statistic, so that the genes with the highest up- or down-regulation by LOXL2 treatment will be at the top or bottom of the sheet, respectively. The selected genes presented in the heatmap are on the basis of function in chondrogenic lineage or relevant to osteoarthritis.

**Figure 2:**
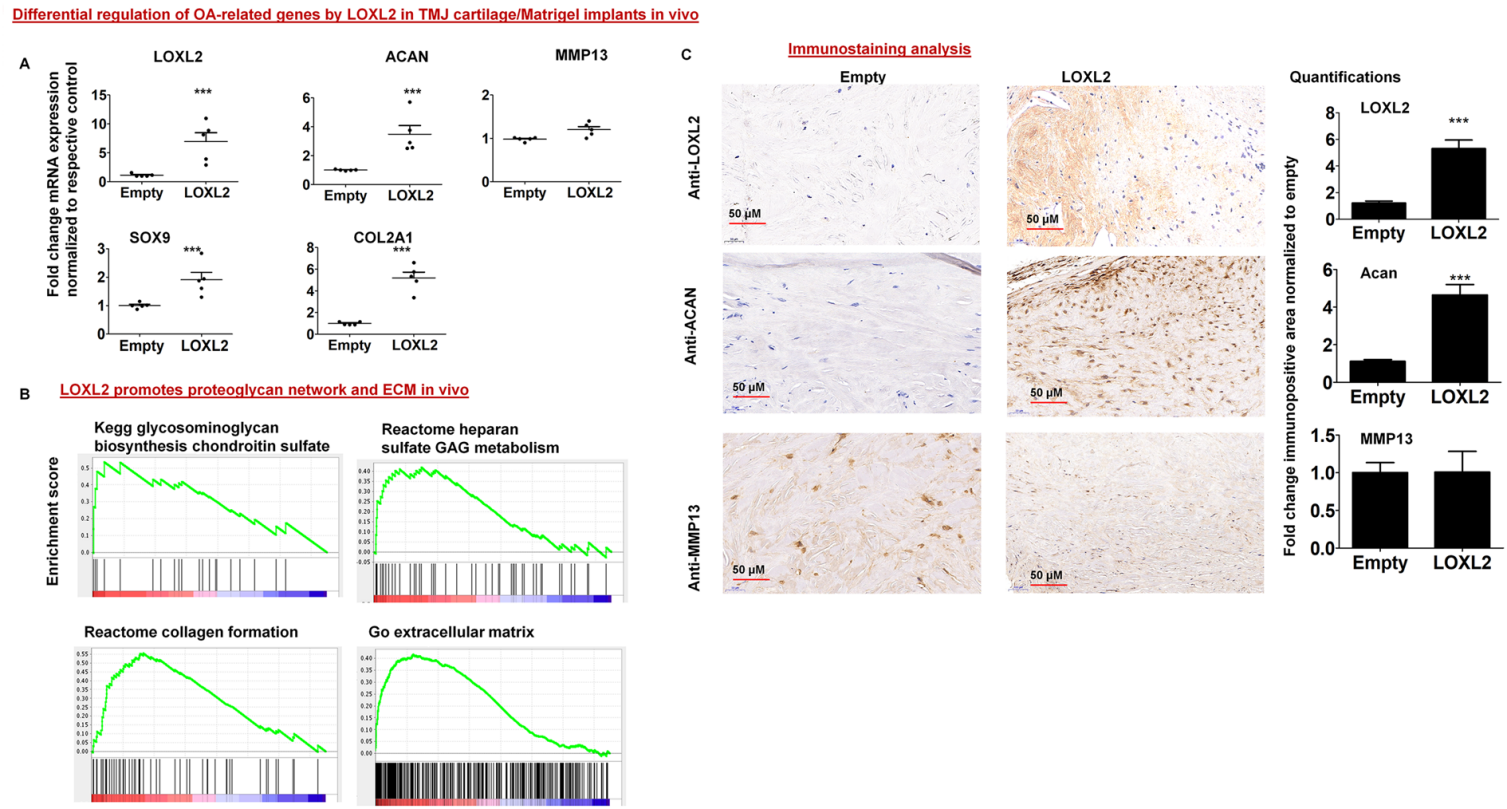
LOXL2 overexpression differentially regulates specific genes and gene networks in human TMJ-OA cartilage implants in nude mice. (**A**) RT-qPCR validation of specific genes in Adv-RFP-Empty and Adv-RFP-LOXL2 treated implants; and (**B**) Gene set enrichment analysis of network regulated by LOXL2. (**C**) The immunostaining and quantification in the extracted implants are shown. The statistically significant differences were evaluated by one-way ANOVA with Bonferroni correction (P < 0.05, **P < 0.01 and ***P < 0.001; ANOVA) is shown.

To evaluate if LOXL2 promotes anabolic responses in degenerative TMJ-OA cartilage, human cartilage/ Geltrex constructs were implanted in immunodeficient nude mice, and cartilage accumulation and gene expression changes were evaluated as a function of LOXL2 adenovirus injections surrounding the implants in vivo. Although various primary human cell-based models have been used^44–46^, we established a new model using superficial TMJ-condylar cartilage and demonstrated the expression of LOXL2 and other pro-inflammatory factors in TMJ-OA. We also used this model was also characterized in our earlier study^19^. The superficial zone of human or animal articular cartilage is enriched with stem cells^47,48^, and of particular interest are the enriched mesenchymal stromal/stem cells (MSCs) in human adult articular cartilage samples from donors aged 47–71-years^49^. Adenoviral transduction of LOXL2 could be detected in TMJ-OA cartilage/ Geltrex implants in vivo (Supplementary Fig. S2). RNA-seq analysis of extracted cartilage implants showed differential regulation of gene expression (Fig. 1). The heatmap of selected genes is shown in Fig. 1, and the complete list is provided as Table 1. The mRNA levels of *ACAN*, and other anabolic genes, analyzed by RT-qPCR and RNA-seq, were higher in cartilage/ Geltrex implants treated in vivo with Adv-RFP-LOXL2 compared to the Adv-RFP-Empty-treated group. The validation of genes by RT-qPCR (Fig. 2A) showed higher expression of LOXL2, ACAN, and COL2A1 in cartilage/Geltrex implants treated with Adv-RFP-LOXL2, compared to Adv-RFP-Empty-injected group, whereas MMP13 gene expression did not change.

**Table 1:** LOXL2-regulated genes in human cartilage/Geltrex implants in nude mice.

Gene symbol(s)	Gene description(s)	Adjusted mean	Fold change	P value	FDR q value
ACAN	Aggrecan	382.6	7.77	0.00001	0.00002
SOD3	Superoxide dismutase 3, extracellular	51.1	4.42	0.00002	0.0001
DLX5	Distal-less homeobox 5	15.8	3.50	0.00002	0.0005
HAPLN3	Hyaluronan and proteoglycan link protein 3	19.6	3.51	0.00002	0.0037
KLF2	Kruppel-like factor 2 (lung)	12.3	2.44	0.0003	0.0185
CHSY3	Chondroitin sulfate synthase 3	45.3	2.10	0.0013	0.0365
SOD2	Superoxide dismutase 2, mitochondrial, nuclear gene encoding mitochondrial protein	189.7	1.84	0.0015	0.0392
HSPA1B	Heat shock 70 kDa protein 1B	10.9	2.38	0.0021	0.0463
SFRP5	Secreted frizzled-related protein 5	20.6	2.62	0.0022	0.0469
WNT5B	Wingless-type MMTV integration site family, member 5B	16.6	2.42	0.0032	0.0548
DLX6	Distal-less homeobox 6	7.2	2.46	0.0041	0.05
BGN	Biglycan	1895.9	1.62	0.0060	0.0695
TIMP3	TIMP metallopeptidase inhibitor 3	930.3	1.70	0.0060	0.0695
HDAC10	Histone deacetylase 10	29.5	2.12	0.0086	0.0812
SDF4	Stromal cell derived factor 4	165.6	1.63	0.0096	0.0847
AJUBA	Ajuba LIM protein	38.9	1.91	0.0115	0.0918
CD97	CD97 molecule	26.5	1.76	0.0206	0.1189
GDF5	Growth differentiation factor 5	4.4	2.04	0.0345	
SOX9	SRY (sex determining region Y)-box 9	15.5	1.53	0.1362	0.3118
COL10A1	Collagen, type X, alpha 1	72.3	1.54		
FGF2	Fibroblast growth factor 2 (basic)	33.8	1.32	0.2781	0.4675
IGF1	Insulin-like growth factor 1 (somatomedin C)	137.1	1.20	0.3947	0.5775
KDM6B	Lysine (K)-specific demethylase 6B	245.0	1.16	0.4195	0.5977
COL2A1	Collagen, type II, alpha 1	3.5	− 1.09	0.000003	0.18
NFKB1	Nuclear factor of kappa light polypeptide gene enhancer in B-cells 1	168.5	− 1.54	0.0342	0.1531
PDCD4	Programmed cell death 4 (neoplastic transformation inhibitor)	327.6	− 2.27	0.0020	0.0455
HDAC2	Histone deacetylase 2	195.5	− 2.01	0.0015	0.0391
CD37	CD37 molecule	57.1	− 2.68	0.0012	0.0361
WNT11	Wingless-type MMTV integration site family, member 11	21.4	− 2.47	0.0008	0.0298
MEF2C	Myocyte enhancer factor 2C	840.9	− 2.75	0.0003	0.0157
CARD11	Caspase recruitment domain family, member 11	92.1	− 3.30	0.0002	0.0156
IRF4	Interferon regulatory factor 4	116.7	− 2.64	0.0002	0.0152
NEB	Nebulin	1985.9	− 5.48	0.00001	0.00002
MYH1	Myosin, heavy chain 1, skeletal muscle, adult	2116.4	− 5.79	0.00001	0.00002
TTN	Titin	17,681.1	− 6.06	0.00001	0.00002

### LOXL2 promotes gene expression networks associated with proteoglycan, collagen and ECM biosynthesis in vivo

GSEA analysis was performed to evaluate human TMJ-OA cartilage/Geltrex implants after Adv-RFP-LOXL2 injection compared with those injected with Adv-RFP-Empty. Transduced LOXL2 promoted gene expression networks involving proteoglycan biosynthetic processes, including those involving the metabolism and biosynthesis of the glycosaminoglycans, chondroitin sulfate, keratan sulfate biosynthesis, and heparan sulfate in vivo (Fig. 2B). Further, LOXL2 promoted networks related to collagen formation, ECM biosynthesis, and epithelial-to-mesenchymal transition (Table 2). Thus, LOXL2 promotes the general upregulation of anabolic gene expression in vivo.

**Table 2:** LOXL2-regulated gene-sets in human cartilage/Geltrex implants in nude mice.

Gene set name	Gene set size	NES	Nominal p value	FDR q value
REACTOME_EXTRACELLULAR_MATRIX_ORGANIZATION	86	2.43	0.000001	*0.0000*
REACTOME_COLLAGEN_FORMATION	58	2.42	0.000031	*0.0000*
PROTEINACEOUS_EXTRACELLULAR_MATRIX	91	2.34	0.00003	*0.0003*
EXTRACELLULAR_MATRIX	92	2.31	0.00001	*0.0004*
REACTOME_INTERFERON_ALPHA_BETA_SIGNALING	49	2.20	0.00001	*0.0020*
REACTOME_CHONDROITIN_SULFATE_DERMATAN_SULFATE_METABOLISM	45	2.11	0.00002	*0.0056*
COLLAGEN	23	2.09	0.00001	*0.0060*
REACTOME_A_TETRASACCHARIDE_LINKER_SEQUENCE_IS_REQUIRED_FOR_GAG_SYNTHESIS	23	2.07	0.00001	*0.0070*
EXTRACELLULAR_REGION_PART	271	1.99	0.00001	*0.0135*
EXTRACELLULAR_MATRIX_STRUCTURAL_CONSTITUENT	25	1.97	0.0027	*0.0141*
SKELETAL_DEVELOPMENT	93	1.97	0.00001	*0.0138*
KEGG_GLYCOSAMINOGLYCAN_DEGRADATION	19	1.96	0.00003	*0.0136*
REACTOME_KERATAN_SULFATE_BIOSYNTHESIS	25	1.96	0.000006	*0.0130*
KEGG_ECM_RECEPTOR_INTERACTION	81	1.95	0.0000008	*0.0132*
REACTOME_HS_GAG_DEGRADATION	20	1.94	0.0025	*0.0134*
REACTOME_GLYCOSPHINGOLIPID_METABOLISM	33	1.93	0.0027	*0.0129*
REACTOME_KERATAN_SULFATE_KERATIN_METABOLISM	29	1.92	0.000001	*0.0142*
EXTRACELLULAR_REGION	352	1.89	0.000001	*0.0181*
KEGG_OTHER_GLYCAN_DEGRADATION	16	1.88	0.0025	*0.0181*
REACTOME_GLYCOSAMINOGLYCAN_METABOLISM	105	1.83	0.000002	*0.0272*
KEGG_GLYCOSAMINOGLYCAN_BIOSYNTHESIS_CHONDROITIN_SULFATE	21	1.81	0.0027	*0.0315*
REACTOME_HEPARAN_SULFATE_HEPARIN_HS_GAG_METABOLISM	49	1.80	0.0000005	*0.0330*
REACTOME_CHONDROITIN_SULFATE_BIOSYNTHESIS	18	1.79	0.0078	*0.0339*
REACTOME_ACTIVATED_TLR4_SIGNALLING	90	− 1.60	0.0054	*0.0204*
REACTOME_MYOGENESIS	27	− 1.63	0.0095	*0.0167*
KEGG_VEGF_SIGNALING_PATHWAY	68	− 1.65	0.0014	*0.0144*
BIOCARTA_INSULIN_PATHWAY	21	− 1.76	0.0030	*0.0060*
REACTOME_DOWNREGULATION_OF_TGF_BETA_RECEPTOR_SIGNALING	21	− 2.16	0.000004	*0.0001*

### Aggrecan and other anabolic genes are regulated in the TMJ of Cho/+ mice by LOXL2

**Figure 3:**
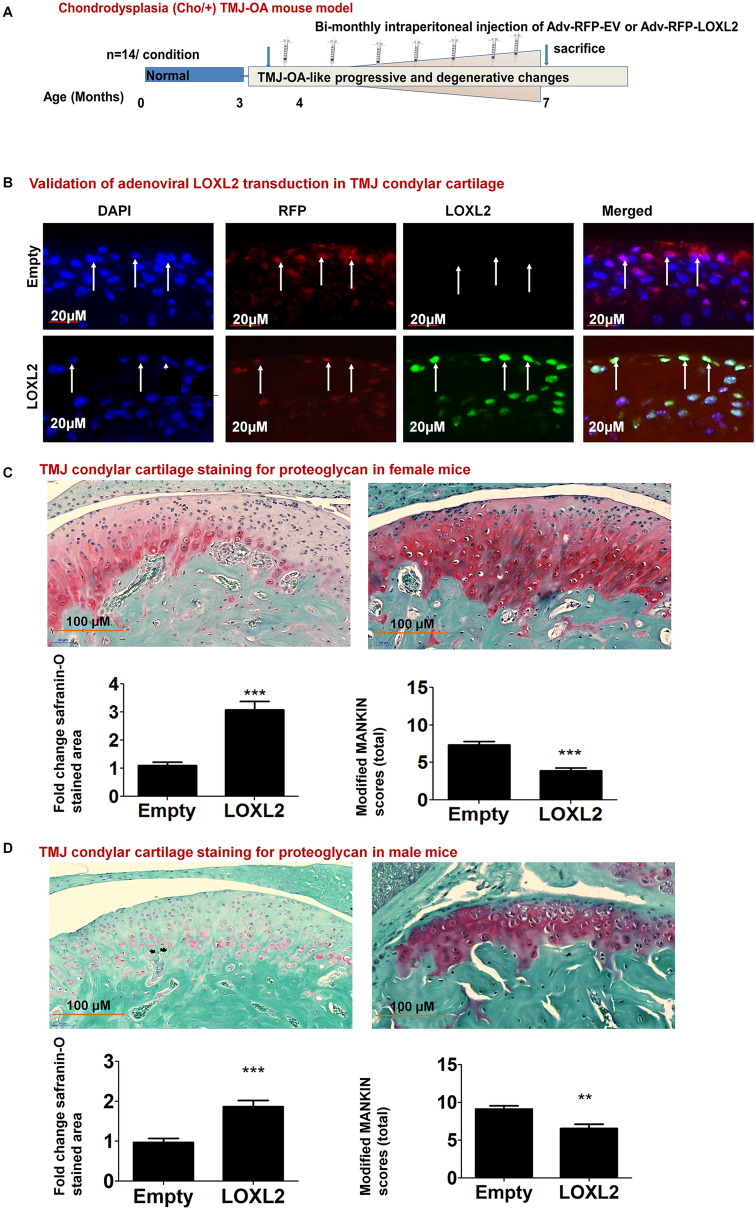
The experimental design in the Cho/+ mouse model and staining of TMJ condylar cartilage. (**A**) Scheme of experimental groups and treatment. (**B**) immunofluorescence analysis for validation of adenoviral LOXL2 expression in TMJ condylar cartilage showing staining for DAPI, anti-RFP and anti-LOXL2 antibodies followed by merged images. (**C**) Safranin-O/Fast green staining of TMJ condylar cartilage in female mice injected with Adv-RFP-Empty compared to Adv-RFP-LOXL2 adenovirus-injected mice, its quantification, and modified MANKIN scoring. (**D**) Safranin-O/Fast green staining of TMJ condylar cartilage in male mice injected with Adv-RFP-Empty compared to Adv-RFP-LOXL2 adenovirus-injected mice, its quantification, and modified MANKIN scoring. The statistically significant differences were evaluated from n = 4–5 TMJ/condition, staining for 5 slides each mice by One-way ANOVA with Bonferroni correction (P < 0.05, **P < 0.01 and ***P < 0.001; ANOVA).

First we validated the differences in wild-type and Cho/+ mice (Supplementary Fig. S2). To evaluate if LOXL2 induces anabolic responses in mouse TMJ progressive OA, Cho/+ male and female mice were used. These mice were injected intraperitoneally with either Adv-RFP-Empty or Adv-RFP-LOXL2 every 2 weeks for 12 weeks, as shown in the experimental design (Fig. 3A). Immunofluorescence analysis demonstrated the successful in vivo transduction of TMJ condylar cartilage by RFP expression and expression of LOXL2 in the respective groups (Fig. 3B). Safranin-O/Fast green staining was increased in the mice injected with Adv-RFP-LOXL2 (LOXL2), indicating restored proteoglycan deposition in condylar cartilage (Fig. 3C). Next, the modified Mankin scoring system was used to assess advanced TMJ-OA histopathology as demonstrated in earlier studies^29,34^. The data from lower and higer magnification images (n = 5mice/ group) were analyzed assigning independent scores such as surface fissuring (0–3), pericellular matrix staining (0–2), the spatial arrangement of chondrocytes (0–3), and interterritorial matrix staining (0–3). The sum of scores of these four criteria for each group were averaged and compared for statistical significance; a higher score indicates more advanced OA histopathology. The inset in Fig. 3D shows the reduction of sum of the scores in Adv-RFP-LOXL2 group.

**Figure 4:**
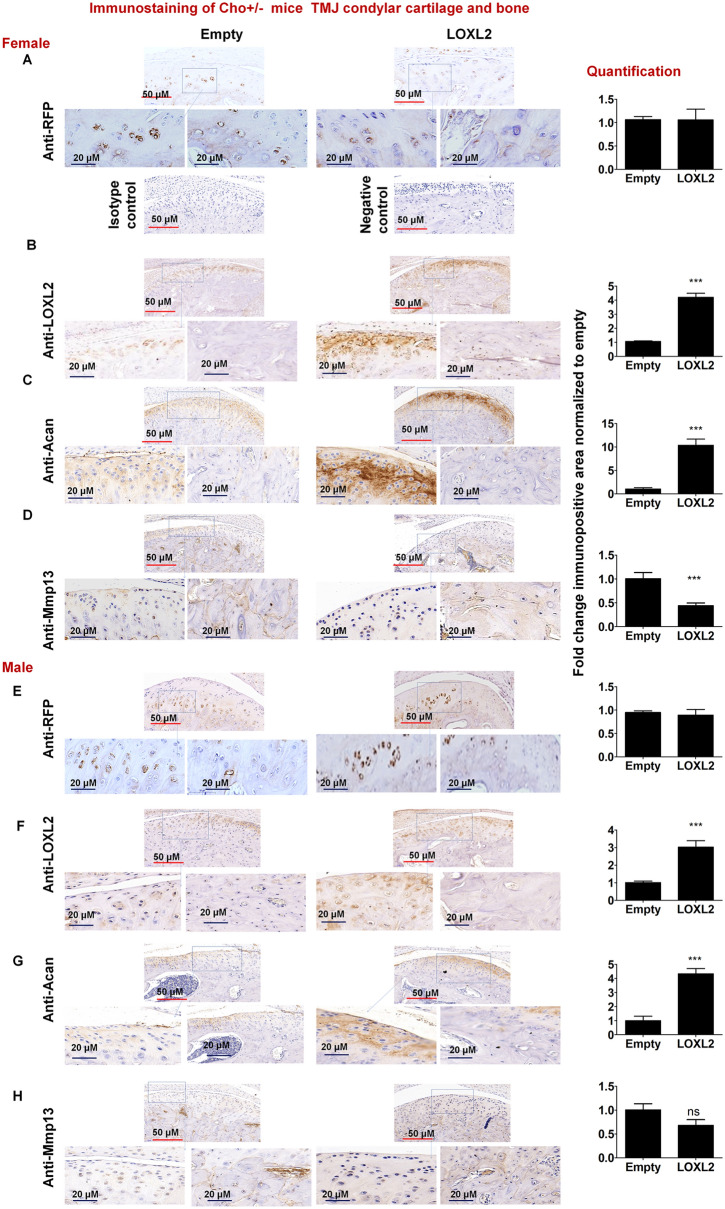
LOXL2 induces a protective response in Cho+/− mice TMJ condylar cartilage. Immunostaining and quantification of (**A**) RFP; (Isotype control is with primary isotype antibody to anti-RFP whereas negative control tissue treated with anti-RFP antibody); (**B**) LOXL2; (**C**) Acan; and (**D**) Mmp13 in Adv-RFP-Empty and Adv-RFP-LOXL2 treated mice with lower (200 ×) and higher magnification (730 ×) of the region of interest from articular cartilage and bone. Immunostaining and quantification of (**E**) RFP; (**F**) LOXL2; (**G**) Acan; and (**H**) Mmp13 in Adv-RFP-Empty and Adv-RFP-LOXL2 treated mice with lower (200 ×) and higher magnification (730 ×) of the region of interest from articular cartilage and bone. The statistically significant differences in immunostaining (n = 3–5/ groups) were evaluated by one-way ANOVA with Bonferroni correction (P < 0.05, **P < 0.01 and ***P < 0.001; ANOVA) for each gene is shown.

Immunostaining analysis of TMJ from female (Fig. 4A–D) and male (Fig. 4E–H) mice injected with Adv-RFP-Empty or Adv-RFP-LOXL2 showed some differences compared to respective control. Immunostaining analysis of RFP demonstrated successful delivery of injected Adv-RFP-Empty or Adv-RFP-LOXL2 to TMJ (Fig. 4A). Two different controls were included for immunostaining; isotype control is with primary isotype antibody to anti-RFP. Figure 4A also includes an image with negative control TMJ from Cho/+ mice untreated (without Adv-RFP-Empty/LOXL2) to demonstrate the specificity of anti-RFP immunostaining. The relative quantification in the cartilage is shown in the right panel. The levels of LOXL2 and ACAN were significantly higher (Fig. 4B–C) and MMP13 was significantly reduced in the cartilage layer (Fig. 4D) in Adv-RFP-LOXL2 group compared to Adv-RFP-Empty injected group. Thus, LOXL2 is protective against the loss of proteoglycan during the progression of OA and may promote increased anabolism throughout the entire joint surface.

**Figure 5:**
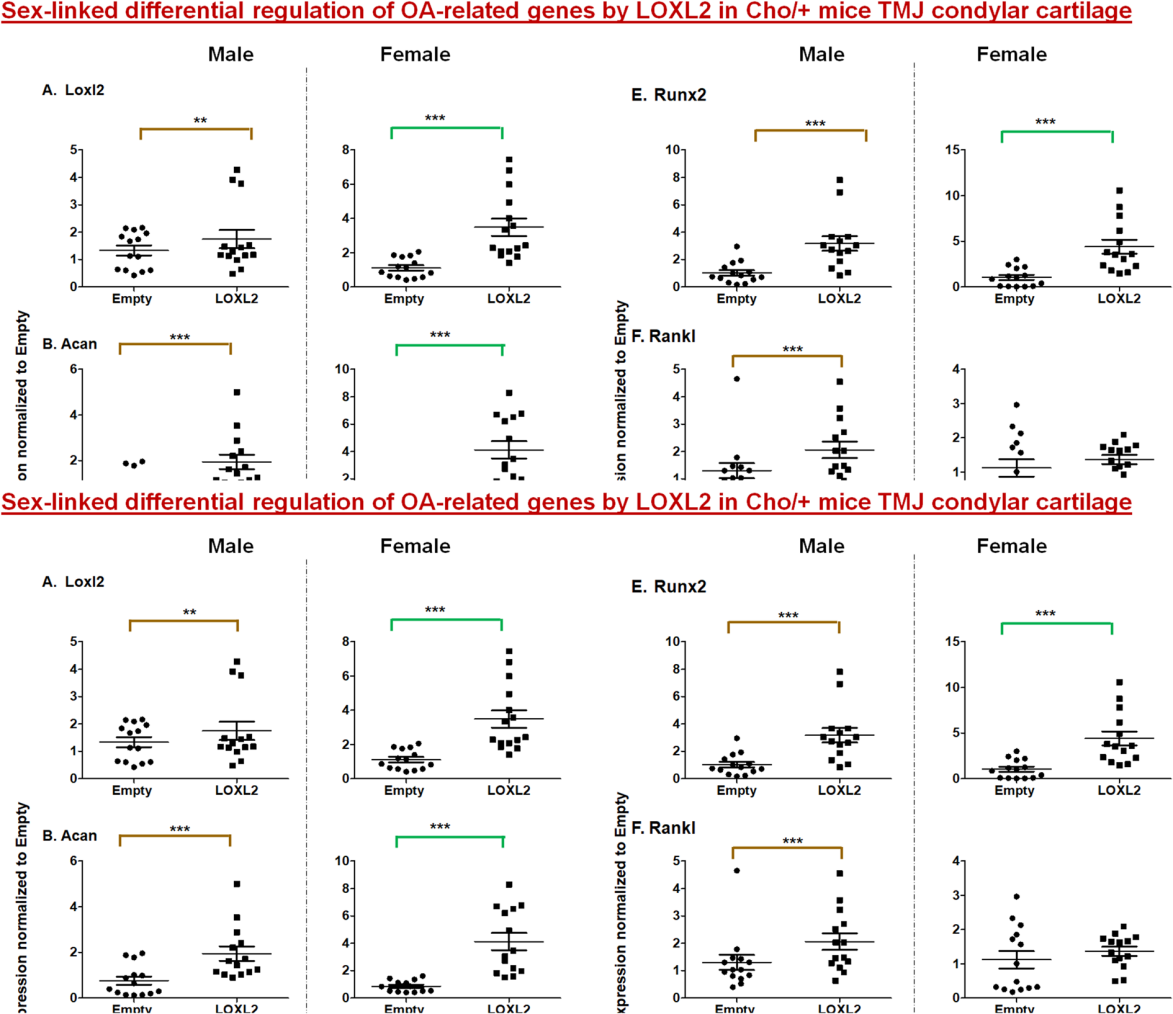
LOXL2 induces mRNA expression of anabolic genes in the Cho/+ mouse model. Each panel shows the fold-change in mRNA levels of differentially regulated genes in Cho/+ mice injected with Adv-RFP-Empty (males and females represented, respectively) and Adv-RFP-LOXL2 (.The statistically significant differences between n = 14 mice/ group (four groups) as Loxl2 (Male) compared to Empty (Male) and Loxl2 (Female) compared to Empty (Female) were evaluated by two-way ANOVA with Bonferroni correction (P < 0.05, **P < 0.01 and ***P < 0.001; ANOVA) for each gene is shown.

Finally, we evaluated gender-specific differences in the group injected with Adv-RFP-LOXL2, in which we detected higher expression of LOXL2 in both males and females, compared to respective controls injected with Adv-RFP-Empty (Fig. 5). LOXL2 significantly increased the mRNA levels of *Acan*, *Sox9*, *Col2a1,* and *Runx2,* indicating that LOXL2 promotes cartilage-related anabolism in general; however, there were differences in the fold-change expression levels in male and females. Interestingly, the levels of *Mmp13* and *Rankl* mRNA were not significantly different in either in male or female mice, comparing those injected with Adv-RFP-LOXL2 or Adv-RFP-Empty. The fold-change levels of *Acan* and *Runx2* mRNA were higher in females compared to males injected with Adv-RFP-LOXL2. Interestingly, Adv-RFP-LOXL2 injection significantly increased *Rankl* expression in males but there was no change in females, whereas *Vegf-B* gene expression was increased in females, but not in males, compared to those injected with Adv-RFP-Empty.

## Discussion

Gene therapy approaches using adenovirus and lentivirus by direct intra-articular joint and intraperitoneal injections have been used extensively in preclinical and clinical studies^50^. The other studies have tested adenovirus-mediated administration of bFGF, IL-1Ra, or IGF-1 and shown efficiency was effective in animal models OA model^51^. Some of the gene therapy approaches are already approved for osteoarthritis^52^. Previous work showed that LOXL2-induced collagen crosslinking enhances the tensile strength of articular cartilage and resistance to collagen proteolysis^53^. During OA, collagen crosslinking is reduced, making the ECM more susceptible to degradation by aggrecanases and collagenases such as ADAMTS4/5 and MMP13, which are the major proteinases that degrade proteoglycans and collagens, respectively, leading to pathological changes in OA cartilage.

Tissues with altered ECM-related proteins have the propensity to develop OA. The LOXL2-induced proteoglycan network in human cartilage implants is critical for maintaining normal joint homeostasis. For example, in TMJ-OA, collagen crosslinking is defective, and collagen is more susceptible to degradation^54^, and a single point mutation in *Col11a1*, as seen in Cho/+ mice^29^, or *Col2a1* deficiency in mice^55^ leads to OA. Intracellular roles for LOXL2 in molecular signaling and epigenetic modification may also affect the development of OA^19,20^. Whether defective LOXL2 function leads to TMJ-OA has not been evaluated.

Our findings in two different in vivo models of TMJ-OA, including human TMJ cartilage/Geltrex implants and Cho/+ mouse, addressed our goal to evaluate if injection of adenovirus-LOXL2 could induce anabolic responses such as *Acan* gene expression in the TMJ. Interestingly, our data showed that MMP13 is downregulated Cho/+ mouse model, whereas higher MMP13 levels and its inhibition by LOXL2 was not observed in human OA tissue implants. In the Cho/+ model, LOXL2 is given prior to manifested pathology, whereas in implant model human TMJ-OA tissues showed advanced TMJ-OA with histologically discernible cartilage pathology. The Cho/+ mouse allowed us to determine that LOXL2 has a beneficial effect in blocking the progression of OA. The implant model allowed us to assess the efficacy of LOXL2 to enhance an anabolic response specifically in human TMJ OA cartilage. Therefore, using a combination of various tools and models, we showed that LOXL2 delivered by adenovirus in vivo produces both chondroprotective and anabolic effects in TMJ. These data have therapeutic implications regarding the potential efficacy of mediators of LOXL2 in treating TMJ-OA.

The TMJ differs structurally from other joints^7^: it contains fibrocartilage, as well as mandibular condylar cartilage (MCC). The MCC distinguishes the TMJ from the articular cartilage of the limbs and cartilage in the cranial base. Differences in the MCC include rapid chondrocyte hypertrophy with the overlapping expression of collagen (COL) types I, II, and X^8^; the absence of growth factors GDF-5 and GDF-6^9^; specific osteopontin and bone sialoprotein expression^10^; and unique responses to gene inactivation^11^. Thus, TMJ cartilage exhibits differences in cartilage structure and protein composition^7–11^ compared to knee and hip articular cartilage and is a more complex tissue. However, LOXL2 induces anabolic responses in both TMJ and knee joint OA, as described in our earlier study^19^.

LOXL2 is a naturally acting enzyme in ECM^56–58^. Moreover, extracellular and nuclear LOXL2 have different functions^59^. In our study, we observed LOXL2 expression in the nucleus after adenoviral delivery, indicating that LOXL2 nuclear function could be responsible for alleviating OA-related changes in Cho/+ mice. We showed that treatment of OA chondrocytes with recombinant LOXL2 or Adv-LOXL2 does not induce proliferation, but only induces differentiation^19^. Taken together, our findings indicate that the administration of LOXL2 could be used as an anti-catabolic or anabolic cartilage therapy in OA.

Since TMJ-OA is a progressive disease, we chose the Cho/+ mouse model, which shows age-related OA changes^29^: (1) at age 6 months, loss of proteoglycan staining at the superficial zones; (2) at 9 months, deficient proteoglycan staining extending from the superficial to the deep layers; and (3) by 15 months of age, typical OA-like joints, including loss of articular cartilage, misshapen meniscus, and inflammation in the synovial tissues, compared to wild type littermates. Thus, Cho/+ mice provide an ideal model for evaluating how LOXL2 extracellular and intracellular functions may protect against OA. Our data showed that LOXL2 induces anabolic responses in TMJ condylar cartilage with progressive OA. Interestingly, LOXL2 treatments produced anabolic responses in male and female mice and demonstrated that LOXL2 could be effective in attenuating OA-related changes in female mice. One limitation in our study is that we did not compare the survival of cartilage implants with and without Geltrex. In the future, it will be necessary to evaluate the effect of Adv-RFP-LOXL2 in implants without Geltrex to determine if its benefits are related to its ability to promote pluripotent stem cell differentiation and support chondrogenesis^21,22^.

Our immunostaining (Figs. 3A and 4A) showed that ip-injected adenovirus LOXL2 directly transduced the outer layer of articular cartilage cells very efficiently (Fig. 4A). The studies have shown that mesenchymal progenitors from synovium, marrow or surrounding tissues are also recruited to the joint structure and articular cartilage^60–64^. Whether these were newly recruited cells from the bone marrow, joint cavity or pre-existing cells is currently unknown. The delivered adenovirus is not very specific with this approach. As delivered, adenovirus could infect TMJ as well as knee joints^19,20^, and other tissues non-TMJ tissues. Interestingly, LOXL2 is a secretory protein, and infection of other non-chondrogenic cell types could lead to an anabolic effect due to circulating LOXL2 protein that targets chondrogenic cells.

In conclusion, our study demonstrates for the first time that adenoviral delivery of LOXL2 induces protective and anabolic gene expression in human TMJ-OA cartilage implants in nude mice and in the Cho/+ mouse model of progressive, age-related TMJ-OA. LOXL2 showed gender-specific differences and similarities in the regulation of anabolic genes. Thus, our findings justify further translational investigation of LOXL2 to determine its potential clinical application for TMJ-OA prevention and therapy.

## Supplementary information

Supplementary information.
