# Supplementary material for: LOXL2 promotes aggrecan and gender-specific anabolic differences to TMJ cartilage
Source: Sci Rep. 2020 Nov 19;10:20179. doi: 10.1038/s41598-020-77178-9 (PMC7678826; doi:10.1038/s41598-020-77178-9)
Supplement: Supplementary file 1 — Supplementary information. [file 41598_2020_77178_MOESM1_ESM.docx]

**LOXL2 promotes aggrecan and gender-specific anabolic differences to TMJ cartilage**

Mustafa M. Tashkandi^1,#^, Saqer F Alsaqer^1,#^, Thabet Alhousami^1^, Faiza Ali^1^, Jennifer Shin^1^, Pushkar Mehra^2^, Larry M. Wolford^3^, Louis C.Gerstenfeld^4^, Mary B. Goldring^5^ and Manish V. Bais^1,*^

**Supplementary Figures:**

**
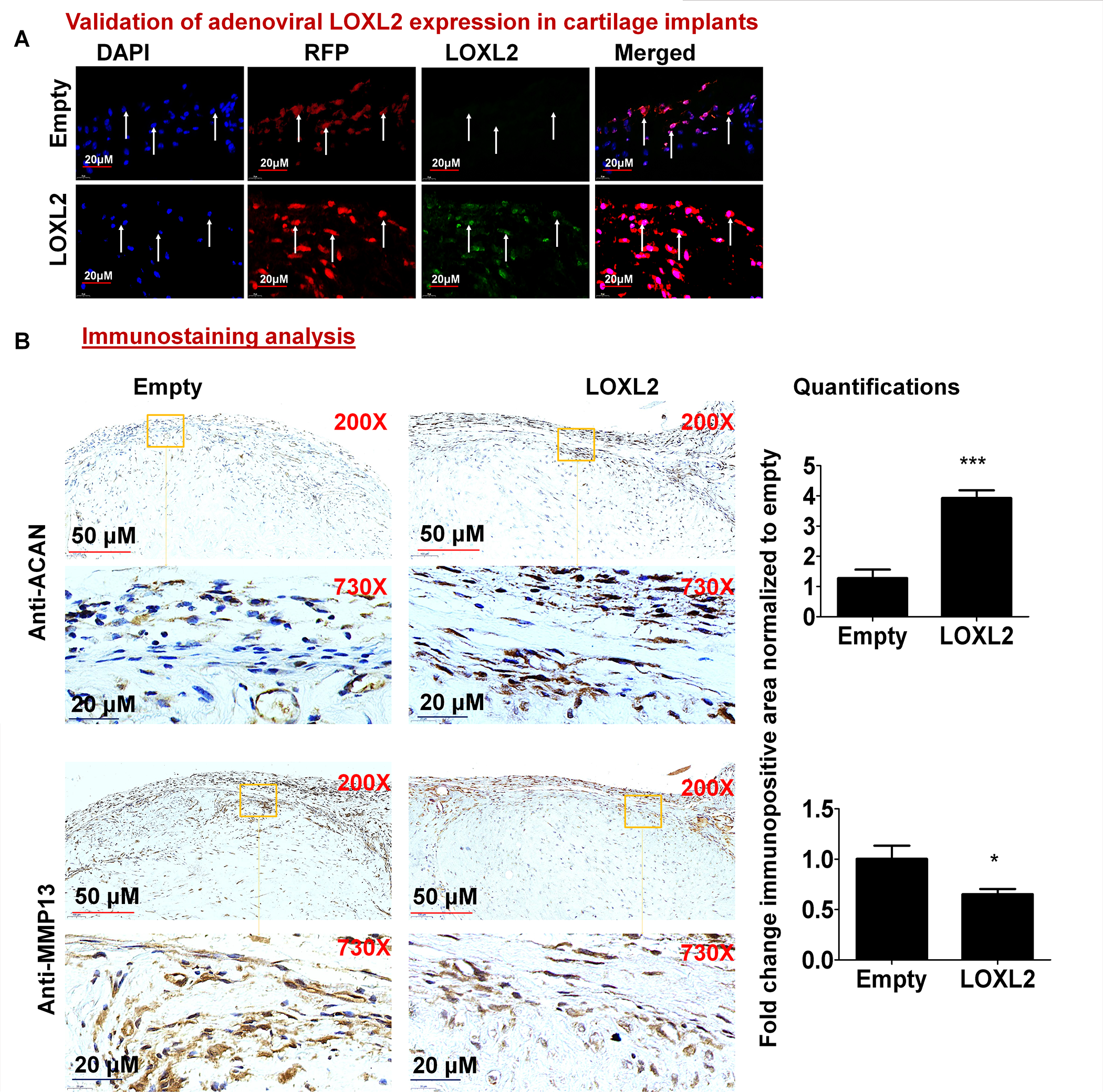
**

**Figure S1. LOXL2 induces the protective response in TMJ cartilage/Geltrex™ implants.** Immunofluorescent analysis of validation of adenoviral LOXL2 expression in TMJ condylar cartilage using anti-RFP, anti-LOXL2 and DAPI, followed by merged images in Adv-RFP-EV and Adv-RFP-LOXL2 treated implants.

**
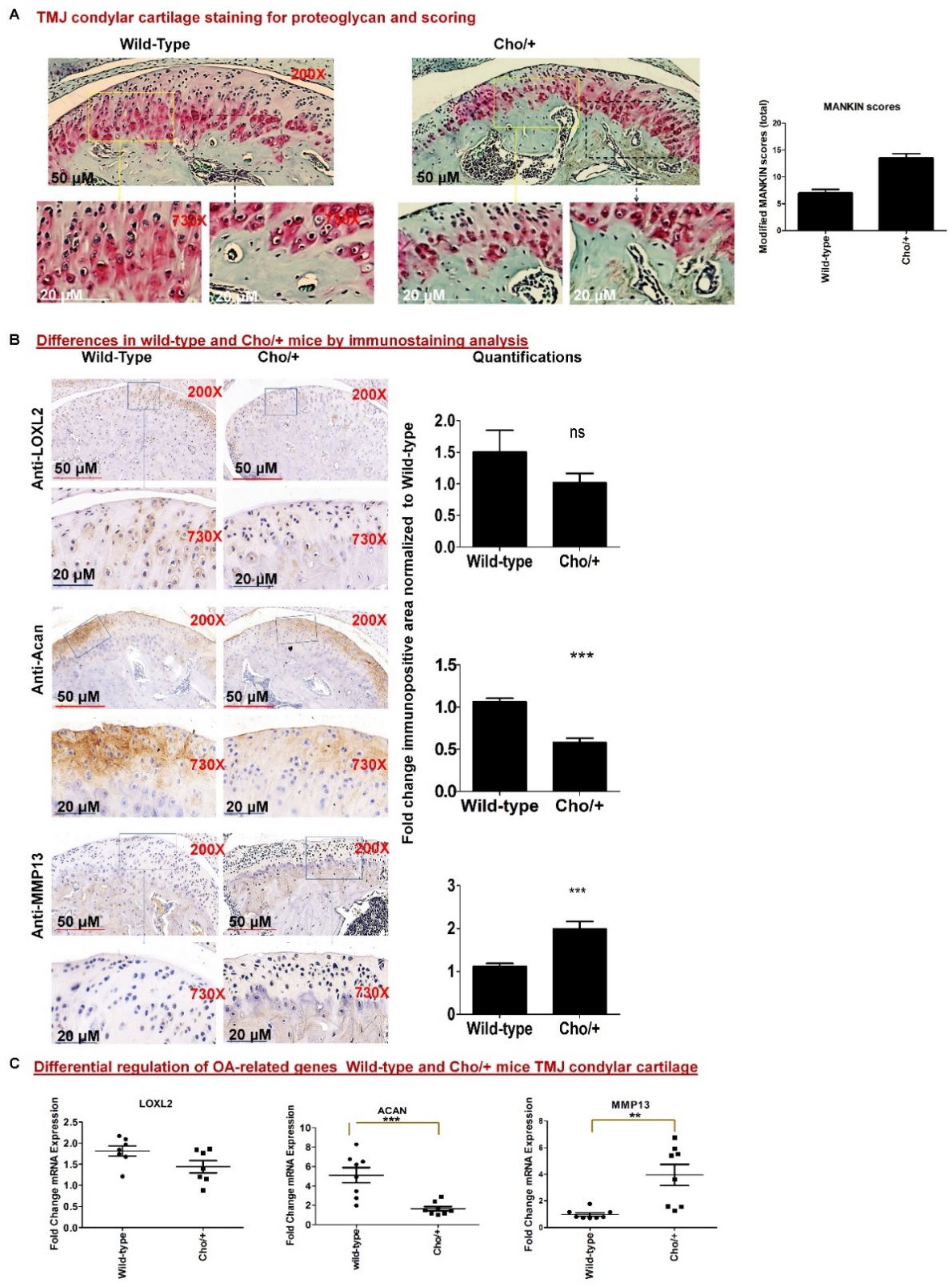
**

**Figure S2. The comparison of wild-type and Cho/+ mice.** A) Safranin-O/Fast green staining of TMJ condylar cartilage in wild-type and Cho/+ mice and its analysis by modified MANKIN scoring. B) Immunostaining and quantification of wild-type and Cho/+ mice TMJ condylar cartilage histology sections for LOXL2, Acan and MMP13 expression with lower (200X) and higher magnification (730X) of the region of interest. The statistically significant differences in immunostaining (n=4/ group; 2 females and 2 males; analyzed independently) were evaluated by one-way ANOVA with Bonferroni correction ( P<0.05, ** P<0.01 and *** P<0.001; ANOVA) for each protein is shown. **C)** Each panel shows the fold-change in mRNA levels of differentially regulated genes in wild-type and Cho/+ mice. The statistically significant differences between n=8 mice/ group were evaluated by two-way ANOVA with Bonferroni correction ( P<0.05, ** P<0.01 and *** P<0.001; ANOVA) for each gene is shown.
